# Infrared nanospectroscopy characterization of oligomeric and fibrillar aggregates during amyloid formation

**DOI:** 10.1038/ncomms8831

**Published:** 2015-07-28

**Authors:** F. S. Ruggeri, G. Longo, S. Faggiano, E. Lipiec, A. Pastore, G. Dietler

**Affiliations:** 1 Laboratoire de Physique de la Matière Vivante, Ecole Polytechnique Fédérale de Lausanne (EPFL), CH-1015 Lausanne, Switzerland; 2 Department of Basic and Clinical Neuroscience, King's College, London, UK; 3 Institute of Nuclear Physics, Polish Academy of Sciences, 31-342 Krakow, Poland

## Abstract

Amyloids are insoluble protein fibrillar aggregates. The importance of characterizing their aggregation has steadily increased because of their link to human diseases and material science applications. In particular, misfolding and aggregation of the Josephin domain of ataxin-3 is implicated in spinocerebellar ataxia-3. Infrared nanospectroscopy, simultaneously exploiting atomic force microscopy and infrared spectroscopy, can characterize at the nanoscale the conformational rearrangements of proteins during their aggregation. Here we demonstrate that we can individually characterize the oligomeric and fibrillar species formed along the amyloid aggregation. We describe their secondary structure, monitoring at the nanoscale an α-to-β transition, and couple these studies with an independent measurement of the evolution of their intrinsic stiffness. These results suggest that the aggregation of Josephin proceeds from the monomer state to the formation of spheroidal intermediates with a native structure. Only successively, these intermediates evolve into misfolded aggregates and into the final fibrils.

## Abstract (web summary)

The onset of neurodegenerative disorders is associated at the molecular level with insoluble protein aggregates, named amyloids. Here, the authors characterize by infrared nanospectroscopy and nanomechanical studies, the amyloid aggregation at the individual species scale.

## Graphical abstract

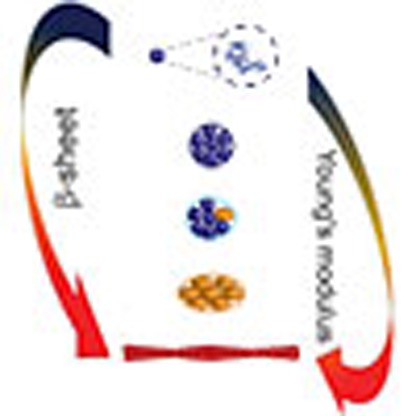

Aging of the population has increased the visibility of several neurodegenerative disorders such as Parkinson's, Huntington's and Alzheimer's diseases^1^. Their onset is connected with insoluble fibrillar protein aggregates, called amyloids^2^. However, these structures are not only associated with diseases since they were also discovered in many physiologically beneficial roles (functional amyloids) including bacterial coatings, adhesives and structures for the storage of peptide hormones^3^,^4^,^5^. During their aggregation, monomeric proteins undergo internal structural rearrangements leading to the formation of fibrils with a universal cross β-sheet quaternary structure^6^. This conformation is independent of the monomeric initial structure and is the fingerprint of amyloids.

The aggregation pathway typically proceeds through the formation of oligomers and protofibrils, which lead to the mature fibrils^7^. Strong evidence indicates that neurodegeneration and cytotoxicity are produced by the intermediate species of fibrillization rather than by the end point. This poses the problem of investigating the early stages of the interconversion of monomers into oligomers and amyloid fibrils^8^. An important question is whether misfolding into β-rich structures precedes or follows aggregation. This is not a trivial point, since the conversion of normally folded proteins into fibrils is generally assumed to be caused by partially misfolded conformations exposing amyloidogenic regions, otherwise protected in the hydrophobic core. A fascinating hypothesis is that not all proteins need to undergo misfolding before aggregation can start. There might be cases in which aggregation proceeds through formation of native aggregates, which undergo misfolding only in an advanced stage. One of the proteins suggested to have this behaviour is the Josephin domain of ataxin-3 (ref. 9). This human protein is of wide interest because its aggregation is responsible for spinocerebellar ataxia of type 3 (refs 10, 11, 12, 13). This hypothesis remains open since, with the currently available techniques, it was impossible to investigate structurally the aggregate intermediate and final species. Its testing requires the use of new characterization methodologies because of the complexity of the energy landscape that leads to aggregation.

Infrared nanospectroscopy (nanoIR)^14^,^15^ is an innovative tool that exploits the combination of two techniques commonly used to study protein aggregation: atomic force microscopy (AFM) and infrared spectroscopy. The first can provide information on the morphology and mechanical properties of the species formed along the aggregation pathway^16^,^17^,^18^, the second can characterize conformational changes in protein secondary structure^19^,^20^,^21^,^22^. Although useful, these conventional techniques do not tell us separately whether/at which time point misfolding occurs, nor what is the correlation between nanomechanical properties and secondary structure of the individual species. Their combination, in nanoIR, enables a structural characterization of the properties of amyloids and the discrimination of objects in the same AFM map by identifying their different secondary structure. Previously, we used nanoIR to analyse patterned lysozyme monomeric and aggregated microdroplets^23^.

In the present work, we employ nanoIR to measure infrared maps and spectra at nanoscale along the whole aggregation pathway of Josephin. We monitor the conversion of monomers into amyloid β-sheet aggregates and link the intrinsic stiffness of amyloid structures to their β-sheet content for the first time at the individual species scale. Our results suggest that misfolding in Josephin occurs only after the first aggregation events. nanoIR can shed light on the structural bases of protein fibrillization and misfolding, allowing a deeper comprehension of the protein aggregation field.

## Results

### Ultrastructural properties of Josephin aggregates

**Figure 1:**
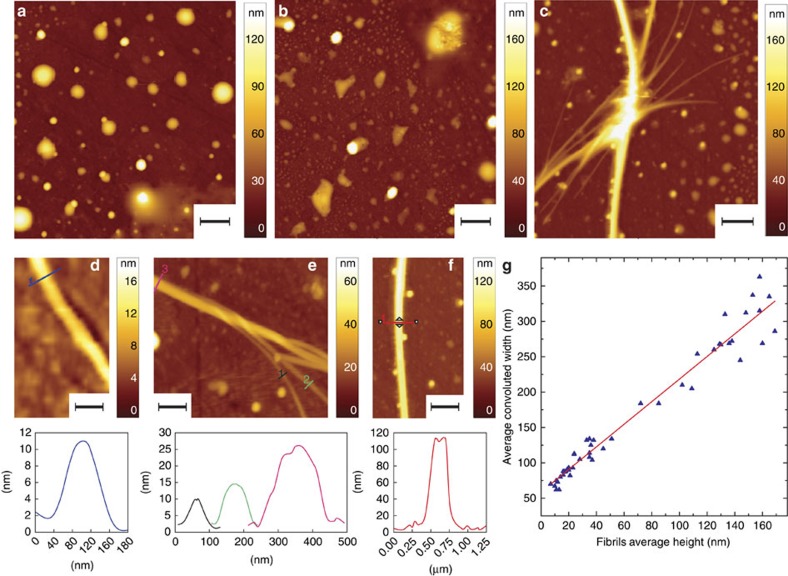
Josephin's aggregation and fibrillization process. AFM morphology at (**a**) 0 day, (**b**) 2 days and (**c**) 7 days of incubation at 37 °C (scale bar, 1 μm). (**d**) Smaller observable fibrils with height in the order of 10–15 nm (7 days, scale bar, 0.5 μm). (**e**) Entangling fibrils with different growing heights (7 days, scale bar, 0.5 μm). (**f**) Supramolecular aggregate with height of ∼115 nm (7 days, scale bar, 0.5 μm). (**g**) Analysis of fibrillar morphology: fibrils' average convoluted width as a function of height. The width convolution effect was caused by the finite lateral dimensions of the tip, which contribute to the measured width of the structures. This unavoidable broadening effect is more important when imaging structures with dimensions smaller or comparable with the tip's apical radius (≈10 nm).

First, we followed the amyloid aggregation of the Josephin using AFM at 0, 2 and 7 days of incubation at 37 °C, to set the scenario for further observations. We studied the samples deposited on a ZnSe surface (Methods). At 0 day, we could observe on this surface monomeric structures with height of ≈5 nm (similar to those evidenced in previous lines of work^9^), spheroidal oligomeric species with height up to ≈50 nm and oligomeric clusters with cross-sectional dimensions up to micrometre size (Fig. 1a and Supplementary Fig. 1). After 2 days, we could still observe a similar picture; however, the oligomer species and clusters lost their spheroidal shape (Fig. 1b). At 7 days, there was massive formation of fibrillar aggregates (Fig. 1c–f). A statistical analysis of the height of the smaller oligomeric species, in the AFM maps on the ZnSe prism, showed that their average height increased as a function of the incubation time during the process of fibrillization. The smaller oligomers had an average height of ∼5 nm at 0 day, of ∼10 nm at 2 days and between 10 and 15 nm at 7 days (Supplementary Fig. 1). The smallest fibrils had an average height of ∼10–15 nm (Fig. 1g). Thus, the height of fibrillar structures corresponded to that of the oligomers. This suggested that aggregation is following the usual process of oligomerization, in agreement with previous studies^9^. Furthermore, the images showed fibrils entangling and forming supramolecular fibrillar aggregates of progressively increasing height and width (Fig. 1d–f), and a quantitative analysis indicated a linear relationship between the height and the convoluted width of these fibrillar structures (Fig. 1g). This suggested that the supramolecular rod-shaped aggregates derive from the association of the isolated population of fibrils with height of 10–15 nm (ref. 24).

To compare the process of amyloid-structure formation on the ZnSe prism with the previous experiments in literature on charged substrates^9^, we performed AFM measurements of the same sample solution deposited on a positively functionalized mica substrate (Supplementary Fig. 2). The results were in good agreement with previous experiments and showed that the use of different substrates enables a wider view of the aggregation process in solution (Supplementary Discussion).

**Figure 2:**
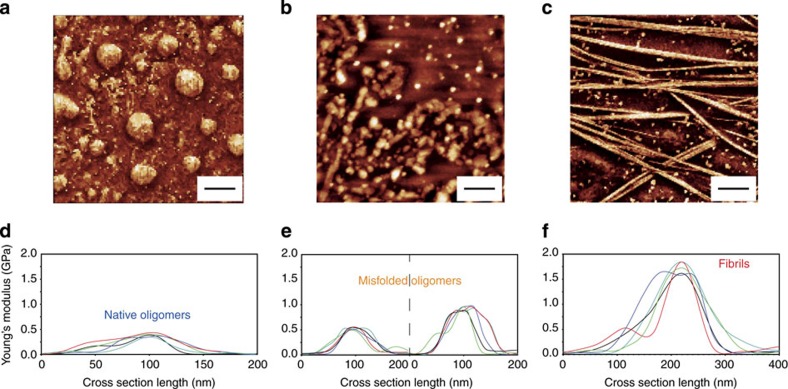
Young's modulus increases as a function of aggregation. AFM quantitative imaging of: (**a**) oligomeric proteins at 0 day, (**b**) oligomers after 2 days and (**c**) fibrillar structures after 7 days of incubation at 37 °C. Scale bar, 2 μm. Stiffness cross-sections of (**d**) oligomers at 0 day, (**e**) oligomers at 2 days and (**f**) fibrillar structures.

To map the evolution of the nanomechanical properties of the Josephin aggregates, we used a fast force–volume system (quantitative imaging (QI)). We produced QI maps of the oligomeric (Fig. 2a,b) and fibrillar (Fig. 2c) structures. Before incubation, the spheroidal oligomers had a uniform Young's modulus of 390±170 MPa (Fig. 2d). This value is appreciably smaller than what expected for an amyloid structure^18^,^25^,^26^. After 2 days of incubation, the sample did not have uniform mechanical properties and two different populations were observed. The first showed stiffness similar to the previous time point (470±150 MPa), while the latter showed a larger Young's modulus of 0.95±0.55 GPa (Fig. 2e), a value consistent with formation of a β-rich structure due to misfolding^27^,^28^. The final fibrillar structures, after 7 days of incubation, had uniform stiffness of 1.70±0.65 GPa (Fig. 2f), in good agreement with the generally accepted value of stiffness of a mature and complete amyloid cross β-sheet structure^18^,^25^,^26^.

### NanoIR of Josephin amyloid species

Although useful, AFM can only provide a morphologic description of the species formed during the fibrillization process but does not tell us whether/at which time point misfolding occurs nor what is the structure of the individual aggregates. Thus, we used nanoIR to characterize further the aggregation pathway. This technique is of particular value when studying non-homogeneous samples, such as amyloid aggregates, offering an important advancement as compared with conventional infrared techniques, which, similar to most spectroscopies, averages the structural information over the highly heterogeneous aggregating solution.

**Figure 3:**
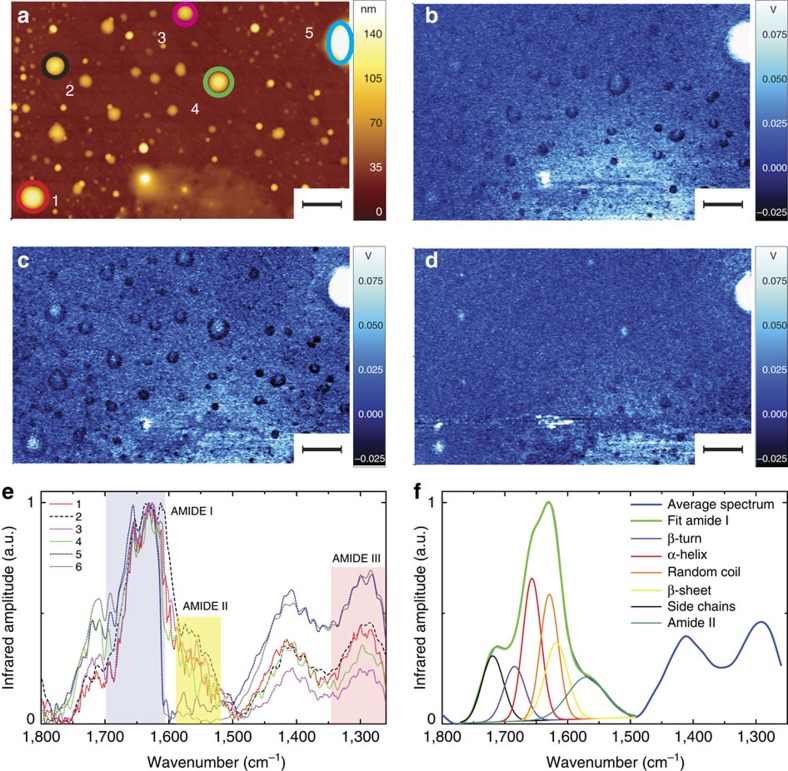
AFM-infrared chemical maps and spectra of Josephin proteins before incubation at 37 °C. (**a**) AFM height image. Infrared absorption map at (**b**) 1,700 cm^−1^ (amide I), (**c**) 1,655 cm^−1^ (amide I), (**d**) 1,300 cm^−1^ (amide III). Scale bar, 2 μm. (**e**) Infrared spectra. (**f**) Average oligomeric infrared spectrum and secondary-structure deconvolution of amide I band.

We measured the structural properties of Josephin before incubation at 37 °C (Fig. 3). The images show the morphology (Fig. 3a) and the absorption of infrared light in the amide I (1,700 and 1,655 cm^−1^, Fig. 3b,c) and amide III (1,300 cm^−1^, Fig. 3d) bands. From the comparison of the absorption maps and considering the components of amide I band related to proteins' secondary structure (Supplementary Discussion), we can infer that the oligomers adsorb mainly infrared radiation in the spectral region of the amide I band, related to random coil and α-helical conformations (1,655 cm^−1^). These chemical absorption maps clearly indicated that the instrument could detect aggregates as small as 50 nm in average height. This height corresponds, for a hemispherical structure on a surface, to a maximum height of ∼100 nm (Supplementary Discussion and Supplementary Figs 3–4). To confirm quantitatively the trend of absorption shown by infrared, we acquired spectra of individual oligomeric aggregates present on the surface (Fig. 3e). This was achieved by positioning the AFM tip on each structure and collecting several spectra inside their area (minimum 10). The comparison of these spectra showed that they had similar peak amplitudes and positions. The amide I band was approximately within 1,655 cm^−1^ (α-helix) and 1,620 cm^−1^ (β-sheet) and centred at ∼1,630–1,640 cm^−1^ (random coil) with a shoulder within 1,720–1,680 cm^−1^ (antiparallel β-sheets, β-turns and side-chain vibrations). The position of the amide II band was approximately within 1,590–1,560 cm^−1^. However, it was not easily measurable because of low signal-to-noise ratio in this spectral range (Supplementary Discussion and Supplementary Figs 4 and5). The amide III band, centred at 1,300 cm^−1^, appeared to be coupled with a second peak, which is visible at 1,410–1,400 cm^−1^. This was already observed in our studies on lysozyme, where we highlighted a change of the relative ratio of the amplitude of this peak and the amide III band during the process of aggregation^23^. This peak can be attributed to a combination of COO^−^, C–N, C–C stretching vibration, C–H and N–H bending, in plane O–H bending. A weaker contribution derives from the vibration of the glutamine (1,410 cm^−1^), glutamic acid (1,404 cm^−1^) and aspartic acid (1,402 cm^−1^) side chains^29^,^30^.

We considered the averaged deconvolution of the amide I band to estimate the secondary structure content of our uniform spheroidal intermediates (Fig. 3f, Supplementary Table 1 and Supplementary Discussion)^19^,^20^,^21^. This analysis provided an estimate of the secondary structure content of 33% α-helix, 29% random coil, 23% β-turn and 15% β-sheet (Supplementary Discussion). These values are in excellent agreement with the monomeric structure of Josephin (35% α-helix, 29% random coil, 19% β-turn and 16% β-sheet) and with previously performed bulk Fourier transform infrared spectroscopy measurements^10^,^31^,^32^. Thus, before incubation, proteins still retain a native structure.

**Figure 4:**
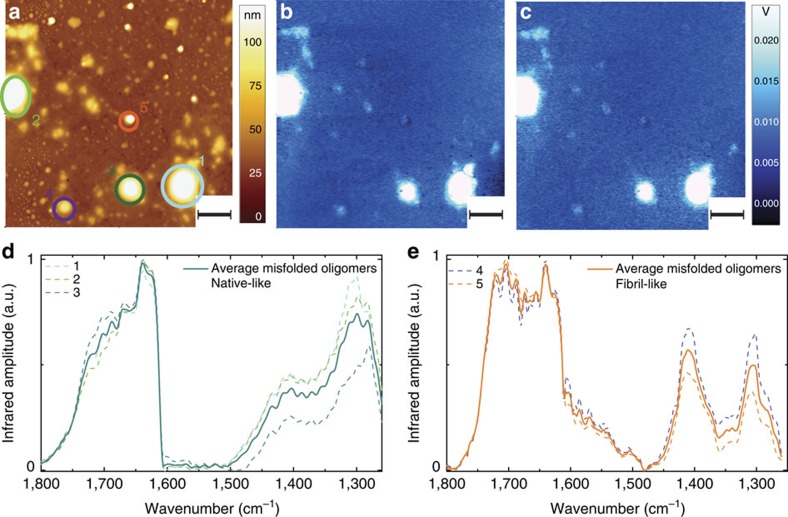
AFM-infrared chemical maps and spectra after 2-day incubation. (**a**) AFM height image. Infrared absorption in amide I (**b**) and at (**c**) 1,700 cm^−1^ and (**d**) 1,655 cm^−1^. Scale bar, 2 μm. (**d**) Spectra of misfolded oligomers, which are native-like with amide I similar to the monomeric structures. (**e**) Spectra of misfolded oligomers, fibril-like showing a conformational switch towards β-sheet structure.

We repeated the measurements after incubation for 2 days (Fig. 4). As for the 0-day case, we collected the AFM morphology (Fig. 4a) and the infrared absorption maps (Fig. 4b,c). The infrared absorption in the amide I spectral region, between 1,700 and 1,655 cm^−1^, remained approximately unchanged. Thus, we focused on the spheroidal structures present on the surface and acquired their full infrared spectrum (Fig. 4d,e). We could distinguish two different families of oligomeric structures. The amide I band of the first oligomeric species resonated at frequencies (1,655–1,620 cm^−1^) similar to the values observed for the spheroidal oligomers observed before incubation. However, the band showed an increased absorption around 1,700 cm^−1^, which is consistent with an increased content of antiparallel β-sheet and β-strand. The shift of the amide I band was more dramatic for the second species, which showed two bands with maxima at 1,700 and 1,640 cm^−1^, respectively, indicating the disappearance of the α-helical component and the increase in the antiparallel β-sheet content. This was also supported by an inversion of the amplitude ratio between amide III band and the one at 1,410 cm^−1^.

**Figure 5:**
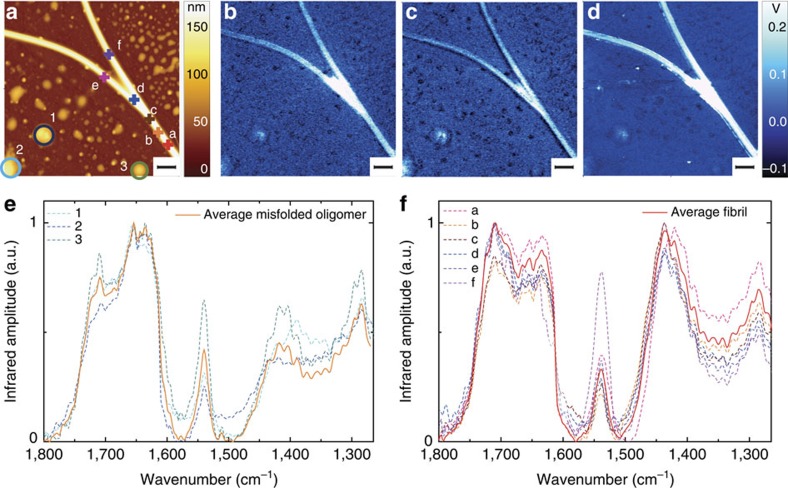
AFM-infrared chemical maps and spectra of fibrils and oligomers after 7-day incubation. (**a**) AFM height. Infrared absorption at (**b**) 1,700 cm^−1^ (amide I), (**c**) 1,655 cm^−1^ (amide I) and (**d**) 1,430 cm^−1^. Scale bar, 1 μm. Spectra of amyloid structures: (**e**) misfolded oligomer (labelled 1, 2 and 3 in **a**) and (**f**) fibrils (labelled a–f in **a**).

After 7-day incubation, both oligomers and fibrils were present in the AFM images (Fig. 5a) with typical average heights of 100–150 nm (Supplementary Fig. 6). Comparison of the absorption maps collected at the amide I band (Fig. 5b,c) and at the band around 1,430 cm^−1^ (Fig. 5d) showed a clear difference between oligomers and fibrils. The first species had higher absorption in the α-helix component of the amide I band (1,655 cm^−1^). The latter had higher absorption close to the β-sheet and β-turn components (1,700–1,680 cm^−1^) and at 1,430 cm^−1^. In other words, by performing absorption maps at different wavenumbers, we could clearly distinguish the structure of oligomers from that of fibrils through their different secondary structure content, which was causing differential response to infrared light exposure.

To prove the behaviour shown by the infrared maps, we acquired spectra of individual oligomeric and fibrillar aggregates (Fig. 5e,f). Overall, the spectra of the fibrils were different from those of the oligomers: they showed a shift of the maximum of the amide I band at 1,700 cm^−1^ and an inversion of the amplitude ratio within the amide III band and the band around 1,400 cm^−1^, which had also a net shift towards higher wavenumbers (∼1,430 cm^−1^).

### Spectral fingerprint of misfolding and amyloid formation

Finally, we performed principal components analysis (PCA) to prove that the spectral differences measured between the different populations were statistically significant and to extract the spectral fingerprint of amyloid formation. PCA allowed noise reduction, resolution of subtle differences and detection of subgroupings within the spectra.

The most important results of PCA are the scores plots and the loadings plots. The scores plots represent the spectra in the multidimensional space of principal components (PCs), which represent the degree of variability within the ensemble of spectra. The loadings plots show which variables are responsible in the data set for the greatest degree of separation inside this spectra ensemble. The spectral differences are presented in the loadings plots. These determine clustering of spectra in the multidimensional scores plots. For the raw data, the maximum (minimum) of loading is related to the position of a particular vibrational motion, which is typical of the spectra clustered on the positive (negative) values of corresponding PC on the Scores Plot.

**Figure 6:**
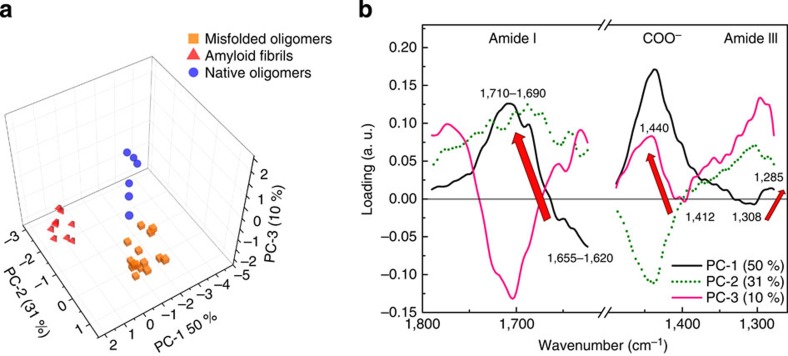
PCA analysis. The results of PCA analysis in the spectral range 1,800–1,270 cm^−1^ (excluding 1,620–1,480 cm^−1^) applied to three groups of spectra: native oligomers, misfolded oligomers and fibrils. (**a**) Three-dimensional scores plot. (**b**) Loadings plot. The arrows indicate the fingerprint of the conformational changes during amyloid formation.

The collected spectra were placed in the space of new independent variables, the PCs. Thus, we could distinguish three distinct clusters, corresponding to three groups of aggregates: native oligomers, misfolded oligomers and amyloid fibrils (Fig. 6a). PC-1, which explains 50% of total variance within the ensemble of spectra (black in Fig. 5b), was positively correlated with the wavenumbers attributed to the amide I in spectral range from 1,710 to 1,680 cm^−1^, to the COO^−^ vibration around 1,430 cm^−1^ and to the amide III at 1,285 cm^−1^ (Fig. 6b). The scores plot showed that these three bands were positively correlated with the PC-1 scores for fibrils and, partially, for misfolded oligomers and negatively correlated with the PC-1 scores for native oligomers and partially for misfolded oligomers (Fig. 6a). This showed that the amide I band between 1,655–1,620 cm^−1^ and the amide III band at 1,308 cm^−1^ are typical of these species. The PC-3, representing 10% of the total variance, clearly indicated a shift of the COO^−^ band. The PC-3 was positively correlated with the COO^−^ band at 1,430 cm^−1^ (fibrils and partially misfolded oligomers) and negatively correlated with the band at 1,412 cm^−1^ (native oligomers and partially misfolded oligomers). We also tested PCA on the second derivatives of the spectra (Supplementary Fig. 7). The conformational change described by the spectra was in good agreement with previous studies in literature using bulk infrared spectroscopy^31^,^32^. During aggregation, the Fourier transform infrared spectroscopy spectrum changed as a function of time, and the main modifications to the amide I band signal were the decrease in random coil and α-helical structure (1,655 cm^−1^), the increase in antiparallel β-sheet content (1,695 cm^−1^) and the decrease in the native β-sheet content. The results achieved using PCA on second derivatives were well in agreement with the analysis of the raw spectra (Supplementary Discussion and Supplementary Fig. 8).

This analysis confirms statistically that the shift of the amide I band from 1,655–1,620 to 1,710–1,680 cm^−1^, the peak at 1,430 cm^−1^ and the shift of amide III band from 1,308 to 1,295 cm^−1^ are markers (arrows in the loadings plot Fig. 6b) of the conformational transitions from the random coil/α-helical structure in native oligomers to β-turn/antiparallel β-sheet conformations in misfolded oligomers and final amyloid fibrils.

### Correlation between nanomechanical and structural properties

**Figure 7:**
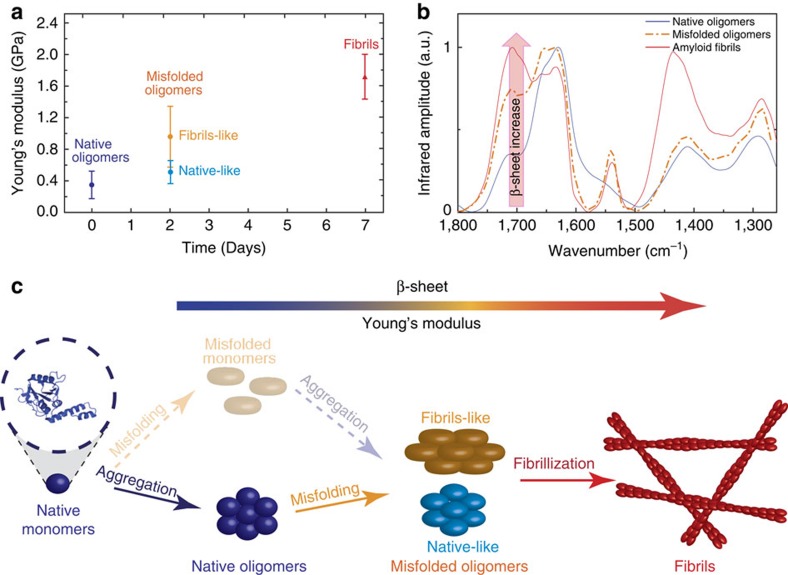
Model of the link between nanomechanical and structural properties. (**a**) The increase in the Young's modulus as a function of fibrillization maturity (the error bars are defined as the s.d. of the distribution of the stiffness values of the aggregates). (**b**) Spectra of native oligomers at 0 day (blue), misfolded oligomers at 2 days (orange and light blue) and amyloid fibrils at 7 days (red). The red arrow indicates the increase in the content of β-sheet secondary structure. (**c**) Model of the possible pathways of Josephin aggregation: the transparent model refers to the generally accepted model ‘first-misfolding-then-aggregation', while the solid one to the new suggested model of ‘first-aggregation-then-misfolding'.

The aggregation pathway of Josephin comprises species of large heterogeneity, as we confirmed by using different AFM-based techniques. Morphology measurements showed that fibrillization follows the usual nucleation process of oligomerization. QI force–volume indicated that the intrinsic stiffness of amyloidogenic species increases as a function of fibrillation maturity (Fig. 7a). A more direct answer on the structural changes that individual amyloid species undergo during aggregation was provided by coupling these results with nanoIR. The initial uniformity of the sample before incubation and the presence of two structurally different oligomeric species at 2 days are well in agreement with the intrinsic stiffness measurements performed using QI. For both techniques, we observed two families of misfolded oligomers (Figs 2e and 4). The first has nanomechanical and structural properties similar to those of native oligomers, and we have denominated it native-like. The latter shows an increased stiffness and β-sheet content, and we called it fibril-like. Finally, the system evolves into a uniform group of fibrillar structures exhibiting the stiffness values and β-sheet content typical of amyloids (Fig. 7b).

## Discussion

Characterization of all the species formed along the aggregation pathway of a protein is essential in view of the role that protein aggregation has in human health and in material science. In this work, we have investigated, for the first time at the nanoscale, the aggregation pathway of the Josephin domain of ataxin-3, a protein known to be highly fibrillogenic. We could observe directly the conformational transition from native spheroidal oligomers, through misfolded oligomers, to the final amyloid fibrils. We probed the secondary structure of the aggregates down to 50 nm and demonstrated that, before incubation, the secondary structure of Josephin inside the spheroidal oligomers is clearly native, while misfolding occurs only later, when a conformational transition towards β-enriched structure occurs. This indicates the intermediate nature of the oligomers, some sharing the structural properties of native monomers (native-like), the others being more similar to fibrils (fibril-like). The fibril-like oligomers form mature fibrils, which have a β-rich structure and mechanical properties typical of amyloids. This means that we can identify the spectral fingerprint of the α-to-β transition that native proteins undergo during fibrillization at the nanoscale.

In this way, we correlated the secondary structure of amyloid intermediates and final aggregates to their nanomechanical properties. Indeed, it is central to measure and quantify the ultrastructural properties of amyloid fibrils to appreciate their full potential as biomaterials. It was already suggested that the content of β-sheets in the aggregates during fibrillization process is an important parameter affecting the nanomechanical properties of these structures. AFM measurements and simulations suggested that the intermolecular interactions (for example, hydrogen bonds) between β-sheet layers are responsible for the notable mechanical properties of amyloid fibrils^33^,^34^,^35^,^36^. Our results directly demonstrated, for the first time at the individual amyloid species scale, that the increase in β-sheet content is a fundamental parameter determining the growth of amyloids' intrinsic stiffness. Moreover, our evidence strongly suggests that Josephin aggregation precedes misfolding, indicating this protein as an example in which the assumption of ‘first-misfolding-and-then-aggregation' may not be true (Fig. 7c)^37^. This possibility is often assumed implicitly or explicitly, mainly because of the observation that misfolding of many globular proteins occurs only on destabilization of the native fold. Such misfolding allows exposure of fibrillogenic sequences otherwise protected in the hydrophobic core. On the other hand, a different pathway to amyloid formation (‘first-aggregation-and-then-misfolding') has been more recently identified, in which aggregation of normally globular proteins has been suggested to start from conformational states close to native ones, with no need of transitions across the major energy barrier for unfolding^38^. Our conclusions agree with the evidence that Josephin aggregation is promoted by exposed hydrophobic patches, which have evolved to allow interaction with the non-pathologic cellular partner ubiquitin.

NanoIR proved to be ideal for this study and to characterize individually the species formed along the aggregation pathway, which comprises species of large heterogeneity. We were able to reconstruct the aggregation process and to link nanomechanical and structural properties of individually amyloidogenic species throughout their fibrillization pathway. Conventional methods could not have provided this information with the same clarity. This is promising for the development of biomaterials for a broad spectrum of applications in food, nanotechnology and in medicine. Under this perspective, the use of nanoIR can have terrific applications and provide a unique tool to study the aggregation pathway of proteins and help us to design molecules that could interfere with amyloid aggregation.

## Methods

### Aggregate preparation

The N-terminal Josephin domain of ataxin-3 (residues 1–182) was produced as reported previously^39^. In brief, Josephin was expressed in *E. coli* BL21(DE3) (Life Technologies, USA) as glutathione *S*-transferase (GST) fusion protein with a cleavage site for the tobacco etch virus protease. The GST fusion protein was purified using a glutathione Sepharose affinity matrix (GE Healthcare, UK) and then cleaved from GST using a recombinant His-tagged tobacco etch virus protease. Final purification was achieved using Ni-NTA agarose (Qiagen, Germany). Josephin was incubated at 37 °C without shaking at a concentration of 14 mM, in 20 mM sodium phosphate, pH 6.5, 10 mM TCEP and 0.025% NaN_3_ (all reagents from Sigma-Aldrich, USA) to follow the aggregation pathway. Aliquots of 10 μl were taken before and after 2 and 7 days after incubation.

### Sample preparation for conventional AFM

We performed conventional AFM measurements in air of the sample deposited on positively functionalized mica. To functionalize the surface, after cleaving, the bare mica substrate was incubated with a 10-μl drop of 0.05% (v/v) APTES ((3-Aminopropyl)triethoxysilane, Fluka) in Milli-Q water for 1 min at room temperature, rinsed with Milli-Q water and then dried by the passage of a gentle flow of gaseous nitrogen. The preparation of the mica AFM samples was realized at room temperature by deposition of a 10-μl aliquot of fully concentrated 14-μM solution for 10 min. Then the sample was rinsed with ultrapure water and dried by a gentle flow of nitrogen. It is fundamental to underline that these last two passages remove all the material on the surface that is not firmly attached.

### QI AFM

We performed all the force–volume stiffness investigations using a JPK Nanowizard III microscope (JPK Instruments AG, Berlin, Germany). The AFM head is working on a commercial inverted optical microscope (Axio Observer.A1, Carl Zeiss, Göttingen, Germany). Analyses were performed using Bruker DNP-10 cantilevers (Bruker probes, Berlin, Germany), choosing the ones with a nominal spring constant of 0.35 N m^−1^. Before each experiment, we calibrated the mechanical properties of the tip using the JPK software. All images were obtained by working in the QI modality, an evolution of the force–volume mode in which the AFM tip is placed in fast oscillation over the sample and the deformation of the cantilever is recorded to reconstruct an image formed by a large number of force distance (FD) curves. Typical images contain up to 256 × 256 pixels and, for every pixel, 2,048 points per FD curves were collected. The length of the curves was 300 nm and the imaging speed ranged from 0.1 to 3 lines per second. The tip–sample interaction was limited to a maximum cantilever deflection of 3 nm (that is, corresponding to a maximum applied force of 1 nN). The data files were recorded on at least five different areas per sample and on minimally 20 different molecules per area. In total, the average values were calculated over >100 individual aggregates. Depending on the size and on the resolution of the image, the stiffness of each molecule was measured on a minimum of five points. Processing was carried out in a semi-automated way with the JPK data-processing software assuming that the cantilever behaved accordingly to the Hooke law (that is, the deflection of the cantilever is directly proportional to the vertical component of the force applied on the tip). In this case, the FD curves collected on the sample can be subtracted from the FD curves obtained on the hard substrate, resulting in indentation curves. For these calculations, we assumed a parabolic approximation of the tip shape. The shape of each indentation curve is then used to calculate the mechanical properties of the sample and specifically the stiffness. The average stiffness values, for each condition (monomers, oligomers and fibrils) in Fig. 6a, are expressed in GPa and we defined the error as the s.d. of the measurements on the different structures we analysed.

### NanoIR measurements

For all the nanoscale infrared measurements, we used a nanoIR platform (Anasys, CA, USA), which combines high-resolution and low-noise AFM with a tunable infrared laser. This instrument allows nanoscale measurements of infrared absorption as a function of the wavenumber to characterize specimens at spatial resolutions not previously achievable. This approach has been made possible by the photothermal induced resonance effect, also referred to as AFM-infrared^40^,^41^,^42^,^43^.

The aliquot samples, diluted at 1 μM, were deposited at the surface of an attenuated total internal reflection element prism made of ZnSe monocrystals (Anasys, CA, USA). The ZnSe surface is hydrophobic, as demonstrated by contact angle measurements^44^. This substrate is worldwide accepted and extensively used to investigate amyloid aggregation and conformational change using attenuated total reflection infrared spectroscopy^21^,^22^,^45^. Then, we dried the samples by evaporation, so that all the proteins in solution were deposited on the surface.

After the preparation, we scanned the samples using the nanoIR microscopy system, with a rate line within 0.08–0.04 Hz and in contact mode. We used a silicon (AppNano, CA, USA) cantilever with a nominal radius of 10 nm and an elastic constant of ∼0.5 N m^−1^. All images have a resolution of at least 1,024 × 512 pixels per line. The AFM images were treated using the SPIP software. The height and infrared amplitude images were first-order flattened. The spectra were collected with a sampling of 1 cm^−1^ and 256 co-averages, within the range 1,200–1,800 cm^−1^ and with a spectral resolution of 8 cm^−1^. Spectra were averaged from at least 10 measurements. They were normalized using the microscope's built-in Anasys software (Analysis Studio) and OriginPRO. Successively, they were smoothed with a Savitzky–Golay filter (second order, nine points).

All measurements were performed at room temperature. The ultimate resolution of the instrument allows detecting objects with thickness of 50–100 nm and with a lateral resolution as small as the radius of the AFM tip. This minimal measurable thickness is defined by the minimal detectable photothermal expansion of the sample. On the other hand, the lateral resolution of an isolated object is only limited by the sharpness of the AFM lever. In the case of non-isolated objects, thermal diffusion can limit lateral spatial resolution.

### Principal component analysis

We performed the PCA by means of the nonlinear iterative partial least squares algorithm with cross-validation and mean-centred data, using the Unscrambler 9.2 software (CAMO, Norway). Analysis was performed on underivatized spectra and second derivatives. Second derivatives were calculated with the Unscrambler 9.2 software using the Savitzky–Golay algorithm for smoothing (second order, 9 smoothing points). The amide II region (1,620–1,480 cm^−1^) was excluded from the analysis because of low signal-to-noise ratio in this spectral range, as mentioned earlier.

## Additional information

**How to cite this article:** Ruggeri, F. S. *et al.* Infrared nanospectroscopy characterization of oligomeric and fibrillar aggregates during amyloid formation. *Nat. Commun.* 6:7831 doi: 10.1038/ncomms8831 (2015).

## Supplementary Material

Supplementary Information Supplementary Figures 1-8, Supplementary Table 1, Supplementary Discussion and Supplementary References
