# Supplementary material for: Infrared nanospectroscopy characterization of oligomeric and fibrillar aggregates during amyloid formation
Source: Nat Commun. 2015 Jul 28;6:7831. doi: 10.1038/ncomms8831 (PMC4525161; doi:10.1038/ncomms8831)
Supplement: Supplementary Information — Supplementary Figures 1-8, Supplementary Table 1, Supplementary Discussion and Supplementary References [file ncomms8831-s1.pdf]

Supplementary Figures

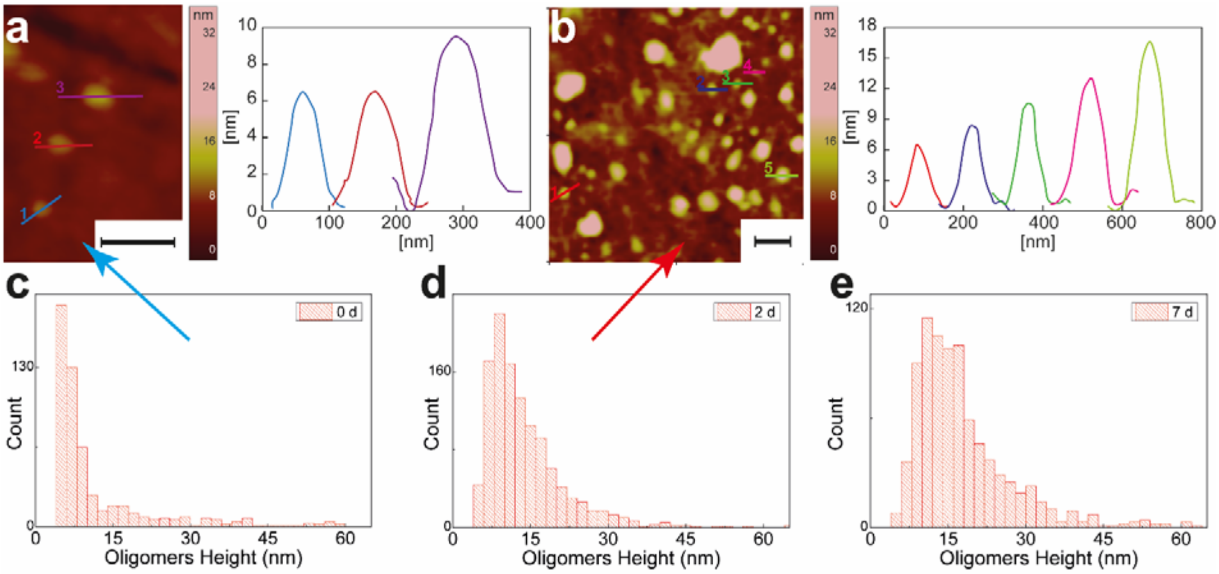

Supplementary Figure 1. Morphological analysis of the oligomerization process on ZnSe prism. AFM detail of the smallest oligomeric species and relative cross-sectional dimensions at a) 0 days (scale bar 0.2 μm) and b) 2 days incubation (scale bar 0.2 μm). Histogram distribution of the smallest height population of oligomers at: c) 0 days, typical value of about 3-6 nm. d) 2 days, typical value increasing to about 10 nm. e) 7 days, increasing height and stabilization of an oligomeric population with approximately 10-15 nm height.

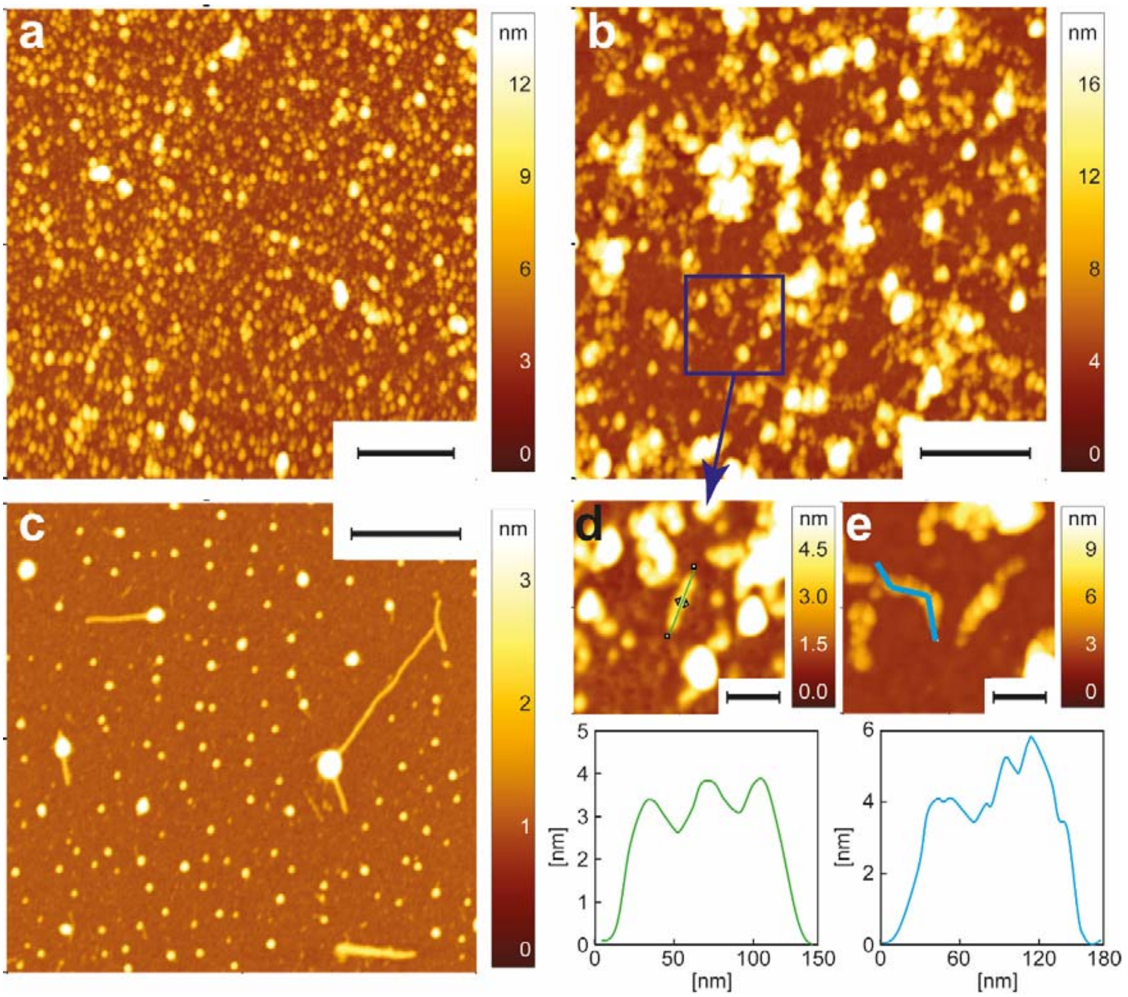

Supplementary Figure 2. Aggregation process on mica substrate.AFM images of Josephin sample on positively functionalized mica at a) 0 days (scale bar 0.5 µm), b) 2 days (scale bar 0.5 µm) of incubation and c) 7 days (scale bar 0.5 µm) incubation. d), e) AFM images (scale bar 0.1 µm) and cross-sectional dimensions of elongated and beaded prefibrillar structures.

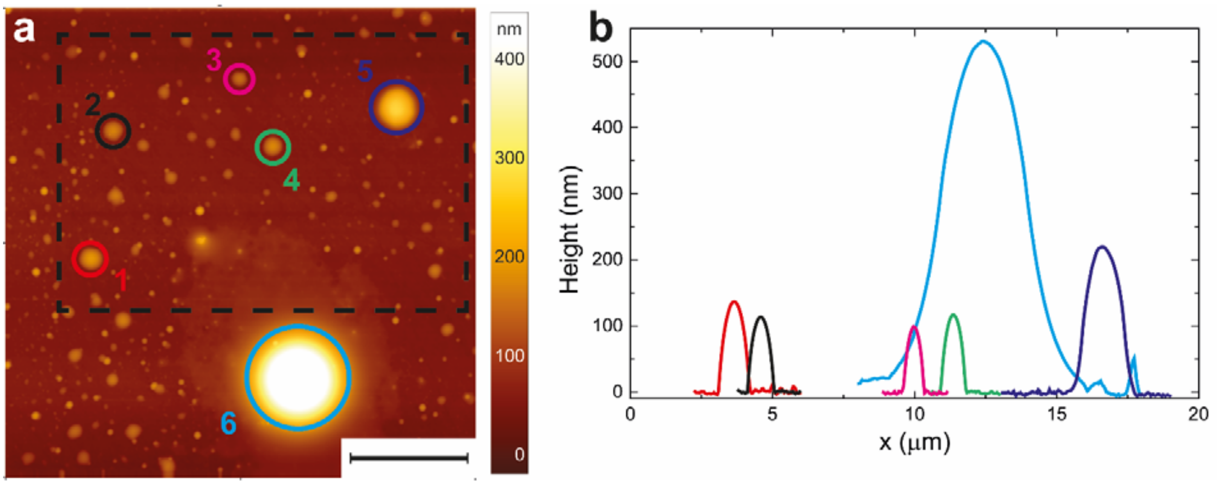

Supplementary Figure 3. Morphological analysis of large oligomeric aggregates at 0 days.
a) AFM height image (scale bar 5 µm). The dotted line approximately represents the area shown in the main text. b) Horizontal cross sections of the structures.

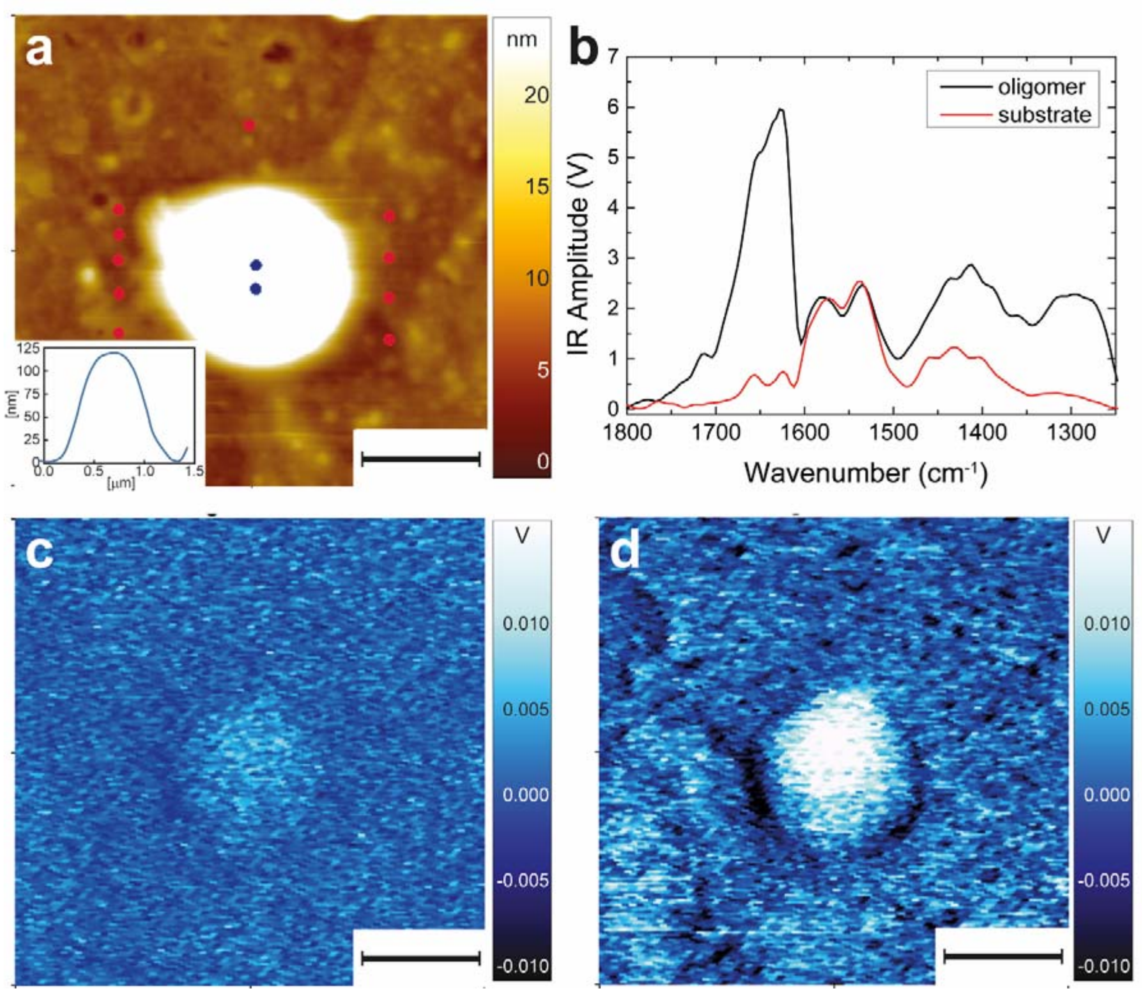

Supplementary Figure 4. **Resolution of nanoIR.** a) AFM morphology map of an oligomeric aggregate with maximum height of 110 nm and average height of approximately 50 nm. b) Comparison of average IR spectra from the top of the amyloid structure (blue dots, 5 spectra per position) and from 10 positions in close proximity to the border of the structure. IR absorption maps in the amide I band at c) 1700 cm^{-1} and d) 1655 cm^{-1}. All images have 0.5 μm scale bar.

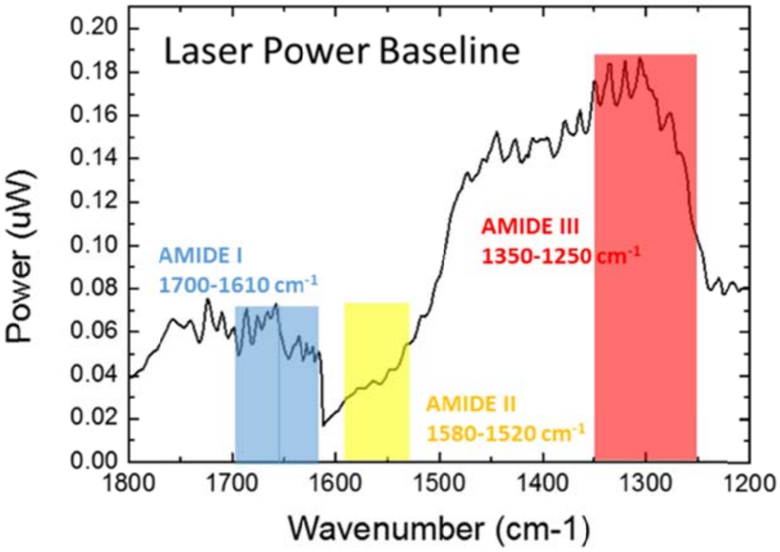

Supplementary Figure 5. NanoIR's laser power spectrum. Effective power of the laser at each wavenumber. The spectra are normalized in order to have constant laser power along all the investigated spectral region.

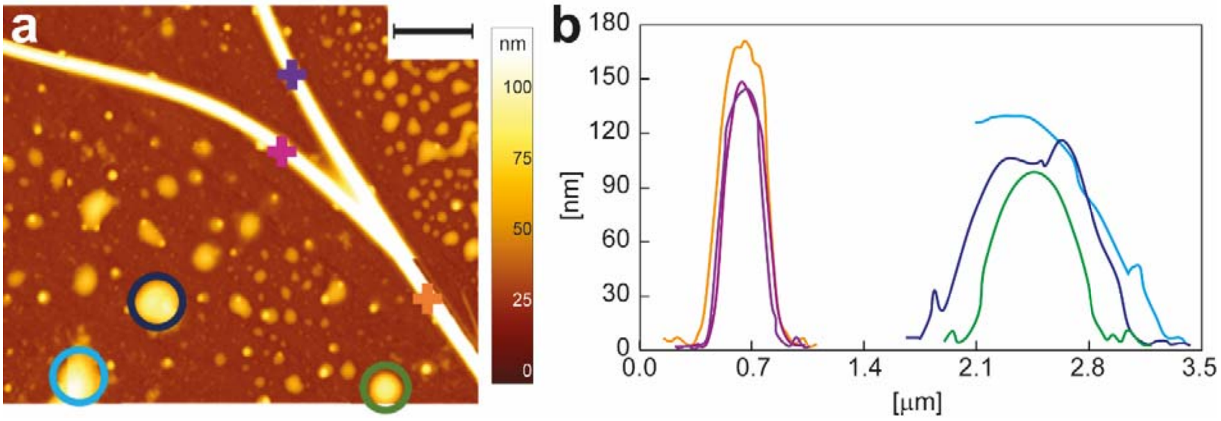

Supplementary Figure 6. Morphological properties of aggregates after 7 days incubation. a) AFM height (scale bar 2 μm). b) Height cross sections.

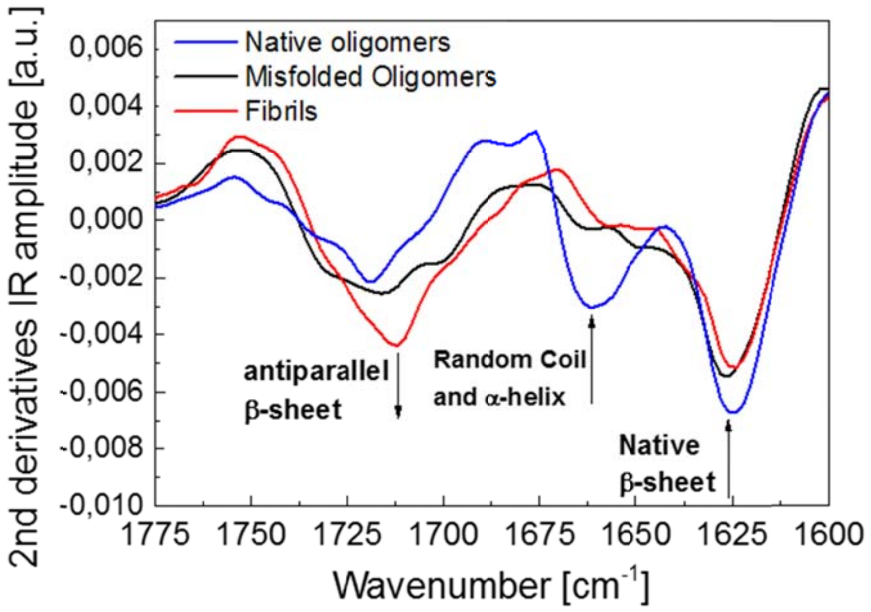

Supplementary Figure 7. Second derivatives of the IR Amplitude. Spectra of native oligomers, misfolded oligomers and fibrillar structures.

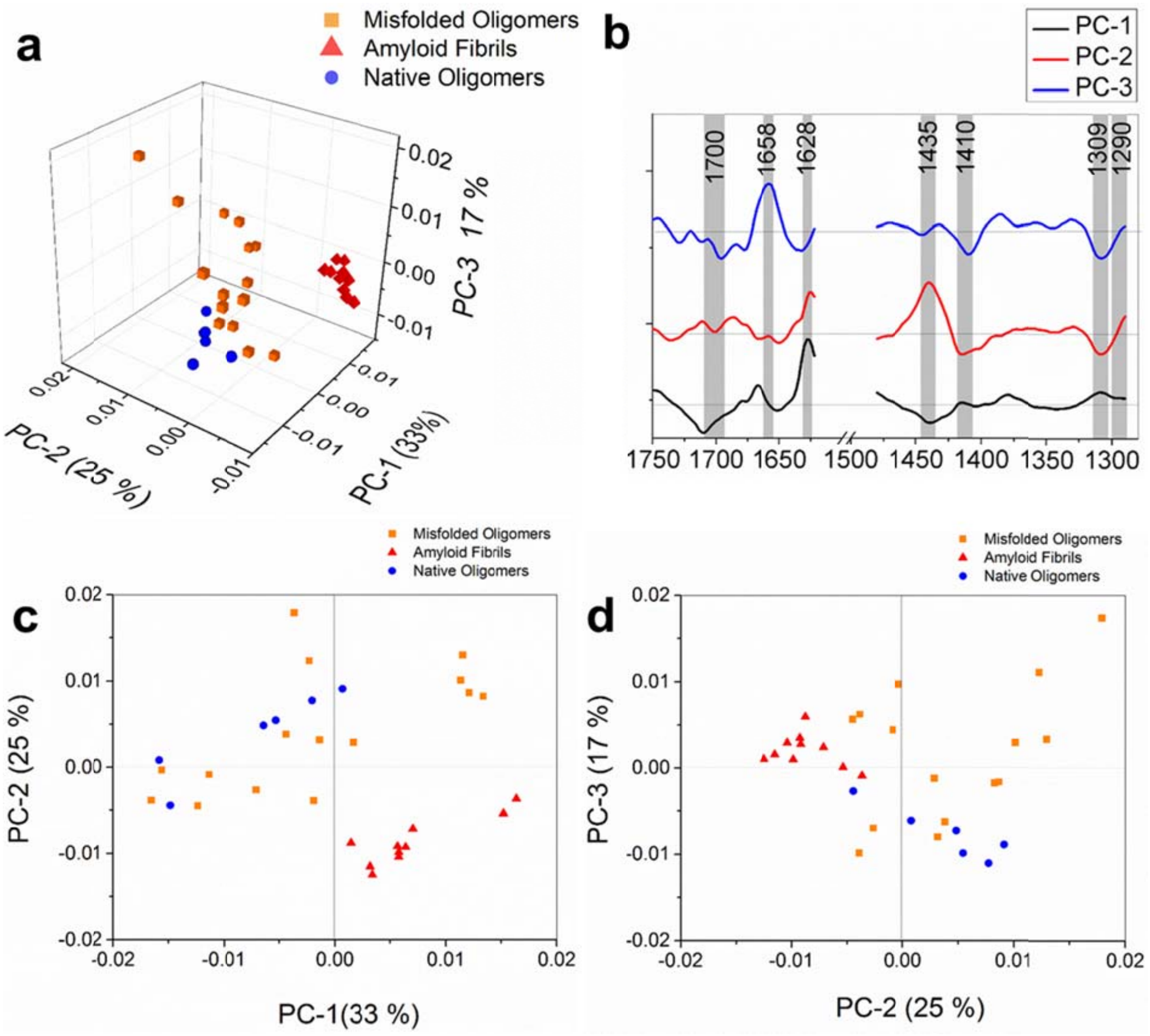

Supplementary Figure 8. The results of PCA analysis applied to the spectral second derivatives. Spectral range within 1800 cm^{-1}– 1270 cm^{-1} (excluding 1620 cm^{-1} – 1480 cm^{-1}) showing: a) 3-D *Scores Plot*; b) *Loadings Plots;* c) PC1-PC2 *Scores Plot*; d) PC2-PC3 *Scores Plot*.

Supplementary Tables

Supplementary Table 1. Amide I Secondary structure components

Amide I Structural assignment	Wavenumber (cm^{-1})
**β-turn and Antiparallel β-sheets**	1695-1665
**α-helix**	1660-1650
**Random coil**	1645-1630
**Low density Native/High density Amyloid β-sheets**	1635-1610

Supplementary Discussion

Comparison of Josephin Aggregation on ZnSe and Mica Substrates

In order to compare the aggregation process on the ZnSe prism (**Fig. 1 and Supplementary Fig. 1**) with the previous experiments in literature on charged substrates,[1] we performed conventional AFM measurements in air of the sample deposited on positively functionalized mica (**Supplementary Fig. 2**).

The AFM measurements on the mica substrate were conducted on the same solution and in parallel with the infrared nanospectroscopy experiments on the ZnSe prism. We investigated the process at the same time points: 0, 2 and 7 days. At 0 days, before incubation, we could observe only monomers and spheroidal oligomers (height ≈30 nm). At 2 days, the images showed interacting oligomers losing their spherical shape and high resolution measurements evidenced flexible and beaded prefibrillar structures. These results were in agreement with what shown by *Masino et al.*[1] Furthermore, the presence of interacting oligomers losing their spheroidal shape is well corresponding with the measurements on the ZnSe prism (**Fig. 1-2)**. Finally, at 7 days, we could not observe stiff fibrillar bundles and aggregates as in the case of the sample deposited on the ZnSe; only low concentrated fibrillar species on mica were present. This is likely caused by the hydrophilicity of the mica surface that did not allow the stable deposition of hydrophobic fibrillar aggregates and bundles. Probably because of a weak sample-surface interaction, these structures do not attach firmly to the mica substrate and that they were rinsed away in the last step of sample's preparation (rinsing by water and flushing by nitrogen).

The morphological differences between structures observed on positively functionalized mica (**Supplementary Fig. 2)** and the structures on the ZnSe prism can be explained by a differential

selective absorption of the amyloid structures in solution by these two surfaces, which possess a different state of charge. Indeed, as shown by contact angle measurements,[2] the ZnSe prism is a hydrophobic surface. Conversely, $MgCl_2$ or APTES treated mica are hydrophilic surfaces with positive charge. This is a fundamental difference when depositing an amyloid aggregating solution on a surface. Indeed, an aggregating amyloid solution is an extremely heterogeneous ensemble of structures with different states of charge. For instance, monomeric proteins of Josephin possess an excess of negative charge (-10), while amyloid fibrillar aggregates are insoluble and highly hydrophobic. The hydrophilicity of the mica surface probably did not allow the stable deposition of large insoluble fibrillar bundles, which were consequently absent on this surface. Whereas, the hydrophobicity of the ZnSe substrate enabled the deposition and the study of the stiffer and hydrophobic fibrillar bundles and aggregates presented in **Fig. 1-2**.

In conclusion, the use of different substrates allowed a wider view of the aggregation process in solution. Indeed, the comparison of the AFM images of the proteins (from the same aliquot solution) deposited on the ZnSe prism and on functionalized mica indicates that fibrillar aggregates are always present in solution and that they are able to attach firmly only on the ZnSe surface.

IR spectroscopy Provides Structural Analysis of the Amyloidogenic Aggregates

IR spectroscopy can be used to observe the conformational transition from monomers to cross β-sheet amyloid structures and investigate their structural properties at the nanoscale during amyloid formation. IR spectroscopy is based on the molecular vibrations produced, under light exposition, by stretching, deformational motions, bending and rotations of chemical bonds. The amide I, amide II and amide III modes are the most commonly used to study the structural properties of polypeptides. The amide I mode arises mainly from backbone C=O stretching

vibrations with frequencies at 1700-1600 cm^{-1}. The exact band position is determined by the backbone conformation and thus by the secondary structure of the protein.[3,4] The amide II band reflects a combination of backbone N-H bending and C-N stretching and has frequencies within 1580-1510 cm^{-1}. The amide III band at 1350-1200 cm^{-1} reflects a combination of different modes such as C-N stretching, N-H bending, C-C stretching and C=O bending. We could associate the position of these two bands to the secondary structure of the protein, but the analysis is far from trivial because of the various contributions. The amide I band is thus the most frequently used to infer the secondary structure of peptides. From several studies, a consensus has emerged for the assignment of the secondary structure IR peaks in this spectral region. [5,6] The components that we have considered concurring to the shape and position of this band are: antiparallel β-sheet and β-turn within 1695-1665 cm^{-1}; α-helical between 1660-1650 cm^{-1}; random coil between 1645-1630 cm^{-1}; low density native β-sheet or high density amyloid β-sheets within 1635-1610 cm^{-1}.

NanoIR principle and sensitivity

The combination of AFM and IR spectroscopy is based on a photothermal induced resonance effect (PTIR). Briefly, if a pulse of IR light at a given wavelength is absorbed by a sample, the local rise in temperature leads to local photo-thermal expansion. This expansion excites the mechanical resonances of the AFM cantilever in contact with the sample. The AFM detection of this temporary dilatation of the scanned region allows reconstructing, together with the acquisition of conventional morphology imaging, the IR-absorption map of the sample and its chemical spectra with a lateral resolution defined in principle by the dimensions of the AFM tip.[7,8] The critical thickness (AFM average height) of the sample needed to acquire IR spectra is

in the order of 50-100 nm and is determined by the ultimate instrument sensitivity to the measurement of the photo-thermal expansion.[8] By acquiring IR maps at different wavenumbers and spectra at specific locations, we could retrieve the spectroscopic properties of native oligomers as small as 50 nm in average height (**Supplementary Fig. 3-4**). This is one of the main advantages of nanoIR compared to conventional IR techniques, which have spatial resolution limited by the smallest achievable IR spot size (~20 μm).[9] NanoIR also requires very small quantities of biological specimens (less than picograms or sub-femtomoles are sufficient).[15] This is a major advantage when working with biological materials whose production is expensive and time costly.

The resolution of the instrument is clearly showed in the **Supplementary Fig. 4**, where we show an AFM image of an oligomeric aggregate (0 days) with average height of approximately 50 nm and maximum height of 110 nm. The comparison of the spectra acquired on the top of the structure and in its close proximity on the substrate clearly demonstrates that we could distinguish the signal acquired from the structure from the residual signal on the substrate, which can be due to the instrumental noise and to the signal deriving from pressure waves of other close features. Furthermore, the spectroscopic behaviour in the amide I band is confirmed by the acquisition of IR absorption maps at different wavenumbers.

Finally, it is worth to note that sensitivity of the instrument in the spectra region within 1610-1550 cm^{-1} is limited by the extremely low laser power in this region (**Supplementary Fig. 5**), which causes low signal-to-noise ratio. This is clear in the **Supplementary Fig. 4**, where the signal on the aggregate is clearly distinguishable from the one on the substrate in the entire spectral range, except in the amide II band. For this reason, in this region, the acquisition of chemical properties of objects with thickness close to the sensitivity limit (average height of 50

nm) is affected by high noise. Thus, spectra of oligomers with average height close to 50 nm and amide II band between about 1590-1560 had extremely low signal to noise ratio. While, in the case of fibrillar structures, the lengths of several micrometres, average heights as big as 200 nm and a shift at lower wavenumbers, where laser power is more intense, allowed to increase the signal to noise ratio in the amide II bands.

Secondary Structure Estimation of Josephin before incubation

We estimated the secondary structure content of Josephin before incubation at 37 ºC by de-convolution of the amide I band of the spectrum of the uniform protein aggregates before incubation (**Fig. 3f**). The analysis was performed by XPSPEAK and a χ-squared of at least 0.001 was reached for the statistical significance of the fit. Although the shape of the band is affected by the high noise between 1610 cm^{-1} and 1550 cm^{-1}, we could estimate a secondary structure content of 33% α-helix, 29% random coil, 23% β-sheet and 15% β-turn.

The shoulder of the spectrum around 1715-1725 cm^{-1} was fitted by considering the IR absorption peaks of the side chain vibrations of aspartic and glutamic acids.[10] The addition of this band in the fit is easily explicable considering that these amino acids are relatively abundant in the Josephin structure (~15% of total composition, ~30% more abundant than in the average protein composition). Moreover, these residues have stronger absorbance coefficients than the average side chains absorption and they are not superimposed to any other secondary structure component of amide I band.

Principal Component Analysis

Unprocessed spectra show pronounced baseline effects. To avoid the possible influence of these baselines on the results and also to present in details the spectral differences related to

the conformational transition of amyloids, PCA was also performed on the second derivatives of the collected data (**Supplementary Fig. 7-8**). In this case, we considered the opposite situation in the interpretation of the correlation between spectra clustering and *Loading Plots* (**Supplementary Fig. 8a-b)**: the maximum (minimum) of loading is related to the position of a particular vibrational motion, which is typical of the spectra clustered on the negative (positive) values of corresponding *Principal Component* (PC) on the *Scores Plot*.

Three clusters of three groups of spectra collected from i) native oligomers, ii) misfolded oligomers and iii) fibrils are well delineated on the three dimensional (PC-1 vs PC-2 vs PC-3) *Scores Plot* (**Supplementary Fig. 8a)**. Two additional *Scores Plots* (**Supplementary Fig. 8c,d**) are shown to visualize the separation along each PC. However, the boundary between a cluster of spectra collected from native oligomers and misfolded oligomers is not clearly evident. This suggests that their conformation is more similar in comparison of the conformation of fibrils. The *Loadings Plot* (**Supplementary Fig. 8b**) indicates that PC-1 (33 % of total variance) is dominated by amide I, which is the most significant marker of amyloid conformational changes. The PC-1 is negatively correlated with an antiparallel β-sheet/β-turn band at 1710 cm^{-1}. This indicates that fibrils (oppositely correlated with a positive PC-1), contain higher levels of antiparallel β-sheet/β-turn conformation in comparison to native oligomers (negative PC-1). In amide I, the PC-1 is positively correlated to the infrared motions corresponding to native β-sheets at 1628 cm^{-1}, which confirms the presence of this conformation in native oligomers. The PC-1 indicates also a shift of the COO- band from ~1435 cm^{-1} (fibrils) to 1410 cm^{-1} (native oligomers). This band dominates the PC-2, showing also a strong positive correlation at 1435 cm^{-1} for fibrils and, partially, for misfolded oligomers (negative PC-2), while for native and misfolded oligomers there is a negative correlation. This is another clear indication that the peak

at 1440 cm^{-1} is typical of amyloid fibrils. The separation along PC-2 can also be explained by the negative loading for PC-2 in the spectral region that corresponds to amide III at 1309 cm^{-1} (oligomers) and positive at 1290 cm^{-1} (fibrils). PC-3 (17 % of total variance) is dominated by the spectral features attributed to α-helix at 1658 cm^{-1}, which is oppositely correlated to all spectra of native oligomers, confirming their high content of this secondary structure, and about 50 % of misfolded oligomers spectra.

Supplementary References

1 Masino, L. *et al.* The Josephin Domain Determines the Morphological and Mechanical Properties of Ataxin-3 Fibrils. *Biophysical Journal* **100**, 2033-2042, (2011).

2 Jeon, J. S., Raghavan, S. & Sperline, R. P. Quantitative analysis of albumin adsorption onto uncoated and poly(ether)urethane-coated ZnSe surfaces using the attenuated total reflection FTIR technique. *Colloids and Surfaces A: Physicochemical and Engineering Aspects* **92**, 255-265, (1994).

3 Kumosinski, T. F. & Unruh, J. J. Quantitation of the global secondary structure of globular proteins by FTIR spectroscopy: Comparison with X-ray crystallographic structure. *Talanta* **43**, 199-219, (1996).

4 Kong, J. & Yu, S. Fourier transform infrared spectroscopic analysis of protein secondary structures. *Acta biochimica et biophysica Sinica* **39**, 549-559, (2007).

5 Haris, P. & Severcan, F. FTIR spectroscopic characterization of protein structure in aqueous and non-aqueous media. *Journal of Molecular Catalysis B: Enzymatic*, (1999).

6 Shivu, B. *et al.* Distinct β-Sheet Structure in Protein Aggregates Determined by ATR–FTIR Spectroscopy. *Biochemistry* **52**, 5176-5183, (2013).

7 Dazzi, A. *et al.* AFM-IR: Combining Atomic Force Microscopy and Infrared Spectroscopy for Nanoscale Chemical Characterization. *Appl. Spectrosc.* **66**, 1365-1384, (2012).

8 Lahiri, B., Holland, G. & Centrone, A. Chemical Imaging Beyond the Diffraction Limit: Experimental Validation of the PTIR Technique. *Small* **9**, 439-445, (2013).

9 Carr, G. L. Resolution limits for infrared microspectroscopy explored with synchrotron radiation. *Review of Scientific Instruments* **72**, 1613-1619, (2001).

10 Barth, A. The infrared absorption of amino acid side chains. *Progress in Biophysics and Molecular Biology* **74**, 141-173, (2000).
